# Functional Bio-Based Additives for Sustainable Polymers: A Systematic Review of Processing and Performance Enhancers

**DOI:** 10.3390/biotech15020031

**Published:** 2026-04-22

**Authors:** Odilon Souza Leite-Barbosa, Debora Cristina da Silva Santos, Cláudia Carnaval de Oliveira Pinto, Fernanda Cristina Fernandes Braga, Marcia Gomes de Oliveira, Marcelo Ferreira Leão de Oliveira, Valdir Florêncio da Veiga-Junior

**Affiliations:** 1 Postgraduate Program in Materials Science and Engineering, Military Institute of Engineering (IME), Rio de Janeiro 22290-270, RJ, Brazil; odilonleitebc@gmail.com (O.S.L.-B.);; 2 Polymeric Materials Technology Laboratory (LAMAP), Materials Division (DIMAT), National Institute of Technology (INT), Rio de Janeiro 20081-312, RJ, Brazil

**Keywords:** bio-based additives, sustainable polymers, plasticizers, flame retardants, circular economy

## Abstract

**Background:** The transition from fossil-derived polymer additives to renewable alternatives is essential to mitigate environmental persistence and ensure chemical safety within the plastics industry. This review provides a comprehensive overview of recent developments in bio-based functional additives and their integration into circular economy frameworks. **Methods:** Following PRISMA guidelines, a systematic literature search was conducted using the Scopus database for studies published between 2023 and 2026. Search terms targeted bio-based plasticizers, flame retardants, antioxidants, and compatibilizers. Studies were screened against predefined inclusion criteria, specifically focusing on experimental validation in polymer matrices, while data mining was employed to map emerging research fronts. **Results:** From an initial 996 records, 54 studies were selected after removing duplicates and ineligible articles. The findings highlight a paradigm shift from passive physical fillers toward active, multifunctional macromolecular agents. Recent literature demonstrates that targeted molecular interventions, such as phosphorylated lignin and biomimetic structures, can resolve trade-offs between ductility and thermal stability at low loadings (<5 wt%). Synthesis routes, performance outcomes, and end-of-life trajectories for each additive class are summarized. **Conclusions:** Bio-based additives have evolved from simple substitutes into strategic tools for the molecular programming of sustainable polymers. Although challenges regarding scalability and high-temperature processing persist, their integration into circular economy strategies establishes a clear roadmap for next-generation bioplastics.

## 1. Introduction

Polymers are indispensable to modern infrastructure, transportation, energy, healthcare, and packaging, due to their unique properties combination of lightweight properties, processability, and a performance-to-cost ratio that enables applications across virtually all industrial sectors [1,2]. In the context of an annual plastic production on the scale of hundreds of millions of tons, with projections for continuous growth, the performance and reliability of these materials have become critical components of global production chains [3]. Consequently, additives are strategic components that modulate rheology, thermal and photo-oxidative stability, flammability, and barrier properties. They not only facilitate efficient processing and extend service life but also allow for the tailoring of polymer properties to meet stringent industrial and consumer requirements [4,5].

Despite their utility, conventional additives, predominantly fossil-derived, such as phthalates, bisphenol A, and various halogenated flame retardants, have sparked significant environmental and health concerns. These substances are linked to endocrine disruption, reproductive toxicity, and potential carcinogenicity [6,7]. Since they are not covalently bonded to the polymer chain, they can easily migrate into food or the environment during the use and disposal of the material. This leaching process is one of the primary mechanisms of contamination, with toxic additives being transported by microplastics into ecosystems. Recent studies on chemical migration in packaging and devices in contact with food and pharmaceuticals show that these additives can release from the polymer matrix over time, reaching the consumer at levels that demand rigorous regulatory control [8,9,10]. In response, regulatory frameworks such as Registration, Evaluation, Authorisation and Restriction of Chemicals (REACH) and Classification, Labelling and Packaging (CLP), specific regulations for food-contact materials, and directives like Restriction of Hazardous Substances (RoHS) have been imposing severe restrictions on families such as phthalates and bromated flame retardants, classifying several of these compounds as Substances of Very High Concern (SVHC) and limiting their use in toys, childcare products, medical devices, and electronics [11,12].

This intensifying regulatory landscape, coupled with the bioaccumulative nature of synthetic additives, drives the industry toward sustainable alternatives with reduced toxicity profiles [7,13,14]. Bio-based additives have emerged as a direct technological response, offering renewable molecules capable of matching or exceeding the functional performance of their fossil-based counterparts. Sourced from plant biomass, agro-industrial waste, or microbial by-products, these additives reduce fossil dependence and lower the carbon footprint of plastic formulations through more benign synthetic routes [15,16]. Furthermore, the integration of bio-based additives with sustainable polymers and biopolymers, such as polylactic acid (PLA), polyhydroxyalkanoates (PHA), and aliphatic polyesters paves the way for the development of systems fully aligned with circular economy concepts, where functional performance, biodegradability, recyclability, and chemical safety are considered holistically in materials design [17,18]. It is important to emphasize that the toxicological profile of a molecule is defined by its chemical structure and not by its origin. Consequently, bio-based “drop-in” additives may also exhibit the same chemical behavior as their fossil-derived counterparts, and any subsequent functionalization of natural precursors must be carefully evaluated to ensure that performance optimization does not compromise safety [19].

Bio-based additives face crucial technical challenges that limit their large-scale industrial adoption. The inherent compositional variability, influenced by plant species, harvest conditions, extraction and purification methods, compromises the standardization of functional properties and compliance with the rigorous specifications demanded by industry [19,20]. Additionally, their frequently inferior thermal stability, high polarity, and susceptibility to oxidation impair integrity during intensive processes such as extrusion and injection molding, while reducing compatibility with hydrophobic polymeric matrices like polyolefins [21,22]. Economic issues further aggravate the scenario, as high procurement costs at scale, the need for complex chemical modifications for optimization, and competition with food supply chains represent persistent barriers to the complete substitution of fossil-based additives [23,24].

Over the last three years, the field has witnessed exponential evolution with multifunctional additives derived from phosphorylated lignin and modified phytic acid, which simultaneously integrate flame retardancy, antioxidant stabilization, and mechanical reinforcement in biopolymers such as PLA and PHA [23,25]. Advances in selective chemical modifications, such as phosphorylation and enzymatic esterification combined with biotechnological routes (fermentation, supercritical extraction) have overcome critical limitations in thermal stability, solubility, and interfacial compatibility. These progressions enable formulations with competitive or superior performance to fossil-based additives, particularly through the development of phosphorus-containing flame-retardant plasticizers derived from L-lactic acid and phytic acid derivatives, along with other recent studies that consolidate the transition to a circular economy [15,26,27].

This change goes beyond simple replacement, integrating bio-based additives into circular economy strategies that prioritize biomass waste utilization, Life Cycle Assessment (LCA), and design for biodegradation. Despite the growing body of literature, previous reviews have become rapidly outdated as scientific publications in this field more than doubled between 2023 and 2026, often failing to capture breakthroughs in molecular programming and multifunctional ‘all-in-one’ agents. To address this and ensure both originality and a focused technical analysis, this systematic review employs a PRISMA-based methodology and bibliometric mapping to resolve recent discordant results regarding performance trade-offs. The scope is specifically narrowed to functional performance additives structured around two critical axes: (i) Processing Aids (including plasticizers, compatibilizers, and lubricants), which optimize rheology and expand processing windows; and (ii) Stability and Reinforcement Agents (flame retardants, antioxidants, and bio-fillers), which ensure thermal durability and mechanical integrity. By focusing on these specific properties and the ‘molecular programming’ era of the last three years, this work provides a high-resolution, updated roadmap for the design of next-generation, industrially viable bioplastics.

## 2. Materials and Methods: Bibliographic Data Analysis

**Figure 1:**
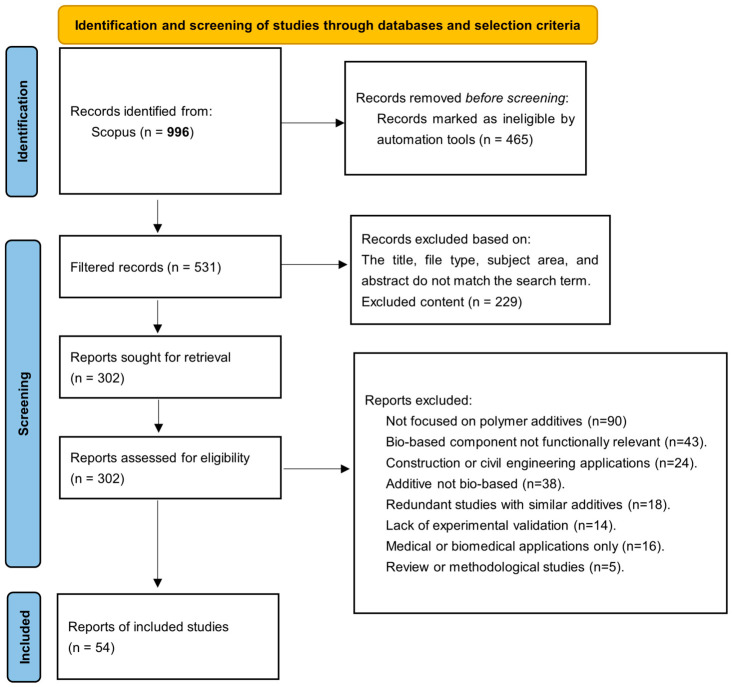
PRISMA flow diagram.

A systematic literature search was conducted in January 2026 using the Scopus database as the primary information source, without the inclusion of grey literature or additional registers. To capture a comprehensive dataset, the search string TITLE-ABS-KEY (“bio-based polymer additives”) was applied and subsequently refined using database filters for timeframe (2023–2026), document type (original research articles), and relevant subject areas. Following this initial filtering, the remaining records were screened by title and abstract to categorize them based on their functional roles, specifically plasticizers, antioxidants, flame retardants, compatibilizers, lubricants, and nucleating agents. Finally, full-text articles were assessed for eligibility, strictly including peer-reviewed experimental studies focused on polymer matrices. Studies were excluded if they: (i) lacked experimental validation, (ii) focused exclusively on medical or construction sectors, or (iii) were non-original research articles. This study adhered to the PRISMA 2020 guidelines, although a formal protocol was not previously registered. The complete selection process is detailed in the PRISMA flow diagram (Figure 1).

Data collection was performed independently by two reviewers using a standardized extraction form to record primary outcomes (thermal stability, mechanical properties, and flame retardancy) and study variables (additive dosage, polymer matrix, and synthesis route). While automation was employed for initial database filtering, data extraction was performed manually by the authors to ensure the precision of the technical outcomes retrieved. The risk of bias was independently appraised by two reviewers, focusing on experimental consistency and adherence to standardized characterization protocols. As this is a qualitative synthesis, results are presented as percentage improvements or absolute value changes rather than through meta-analysis. Studies were grouped by additive functionality, and data were tabulated to compare performance across different matrices. Missing data were managed by prioritizing primary experimental results, and no sensitivity or certainty assessments were performed due to the fundamental nature of the materials science studies reviewed.

To ensure a comprehensive bibliometric mapping, the retrieved metadata (Title, Abstract, and Keywords) were exported and analyzed using VOSviewer (version 1.6.20, Leiden University, Leiden, The Netherlands). A keyword co-occurrence analysis was performed using the full counting method to identify emerging thematic clusters and the evolution of the field since 2023. A thesaurus file was applied to normalize synonyms, such as converting ‘bio-plasticizer’ to ‘plasticizers’, and to unify spelling variations.

## 3. Results

### 3.1. Updated Bibliometric Landscape of Bio-Based Polymer Additives

**Figure 2:**
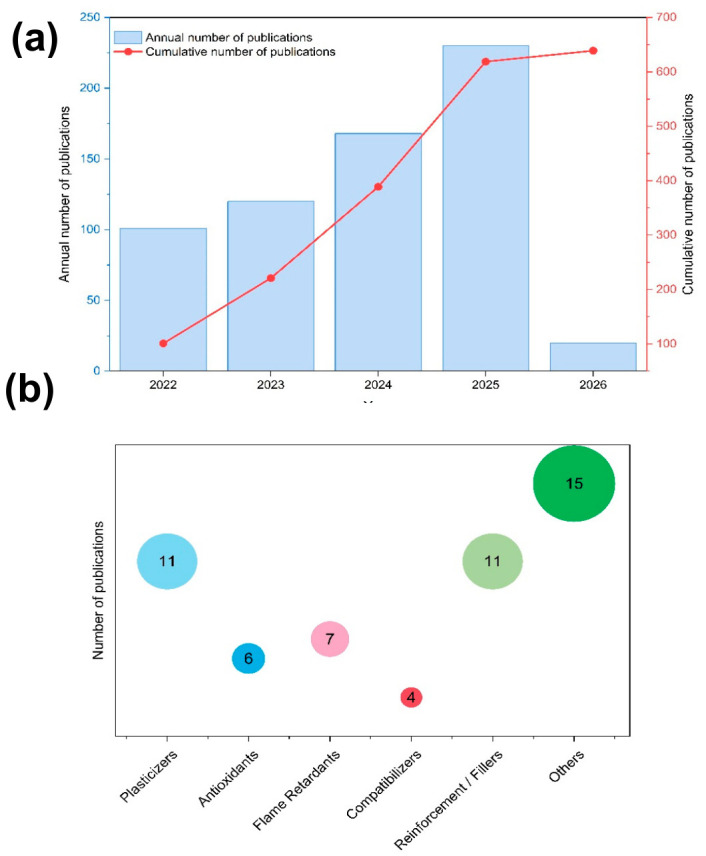
Bibliometric overview of research on bio-based polymer additives. (**a**) Annual and cumulative publications (2023-January 2026). (**b**) Distribution by functional additive category; bubble size and numbers indicate the number of publications. Data for 2026 correspond to a partial year.

An analysis of the current literature (2023–2026), employing the same search criteria, (“bio-based polymer additives”), as Marturano et al. [15], reveals an exponential growth in research output. While the previous study reported a peak of approximately 80 documents in 2021, annual publications have more than doubled, reaching 208 in 2025 (Figure 2). This surge confirms that sustainable plastic formulations have transitioned from a niche academic interest to a dominant frontier in materials science.

**Figure 3:**
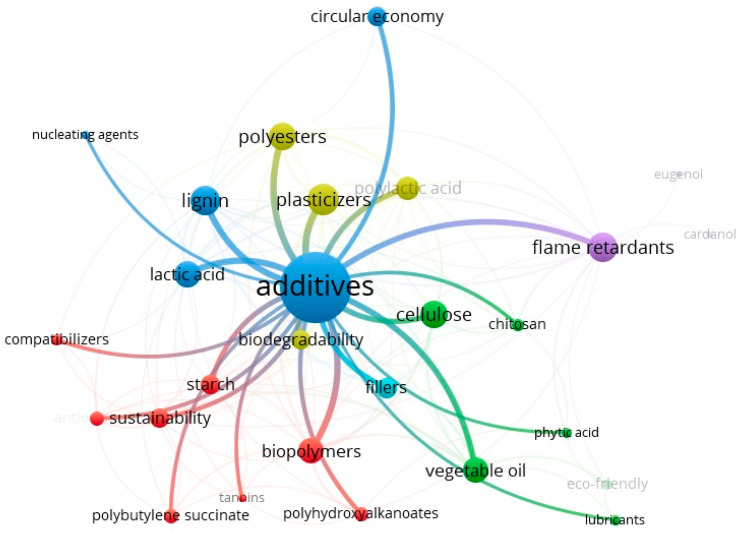
Bibliometric network visualization map of terms related to bio-based additives for polymer processing. The different colors represent distinct research clusters, grouped by co-occurrence strength, illustrating the main thematic areas in the current literature. The lines indicate the connections between the terms, and the node size reflects their frequency of occurrence.

The VOSviewer network map (Figure 3) illustrates the research landscape by highlighting key thematic clusters based on term co-occurrence. The central ‘additives’ node acts as a primary hub, heavily linked to ‘plasticizers’ and ‘flame retardants’, which belong to distinct clusters (yellow and purple, respectively), confirming them as the most extensively investigated functional classes. A significant cluster (red nodes) groups biopolymers such as polyhydroxyalkanoates (PHA) and polybutylene succinate (PBS) with compatibilizers, reflecting the critical role of interfacial engineering in sustainable blends.

Concurrently, the green cluster highlights the emergence of bio-based precursors and processing aids, where vegetable oils and phytic acid signal a definitive shift away from traditional halogenated compounds toward renewable chemical platforms. In the blue cluster, nodes for lignin and lactic acid occupy strategic bridging positions, connecting molecular precursors to circular economy frameworks and biodegradability. Specifically, lignin’s central position underscores its multifunctionality, acting as both a structural reinforcement and a stabilizer. The proximity and strong interconnections between these functional nodes and environmental terms demonstrate that modern additive development has transcended mere performance optimization, becoming deeply integrated into the lifecycle and chemical safety requirements of the circular economy.

The 54 included studies were subjected to the risk of bias assessment described in the methods section. All selected articles demonstrated high experimental reliability, characterized using standardized testing (ASTM/ISO) and consistent characterization protocols. No significant reporting biases were identified, although the certainty of evidence for industrial scalability remains limited by the laboratory-scale nature of the primary data.

### 3.2. Recent Advances in Functional Bio-Based Additives

The rapid evolution of bio-based additives has yielded a diverse array of molecular architectures, ranging from discrete small molecules to complex, heterogeneous macromolecular networks. To elucidate the structure-property relationships discussed herein, Table 1 provides the chemical nomenclature and structural representations for selected additives that exemplify current design trends.

These molecules were selected for their well-defined chemical identities, which facilitate the visualization of reactive moieties, such as phosphaphenanthrene groups, aromatic scaffolds, ester linkages, and furan rings, responsible for their functional performance. Notably, while this review also discusses promising biomass-derived materials like lignin and agro-industrial residues, their high polydispersity and inherent compositional variability make them more accurately described by their functional group density rather than a single idealized formula. Thus, Table 1 establishes a foundational visual framework for the discrete agents increasingly employed to ‘program’ the properties of next-generation sustainable polymers.

As illustrated in Table 1, the structural diversity of these agents is the primary driver of their multifunctionality. The precise arrangement of groups, such as the phosphonate units in U-DC or the dioxime symmetry in DFFD, dictates the additive’s ability to simultaneously enhance processing, stability, and end-of-life performance. The following subsections detail how these specific chemical features translate into functional improvements, shifting the paradigm from simple physical fillers to targeted molecular interventions.

#### 3.2.1. Bio-Based Plasticizers

Recent studies emphasize waste valorization from agro-industrial residues as a central strategy for the circular economy in biobased plasticizers for biodegradable polyesters, to overcome processing and rigidity limitations. Togliatti et al. [28] exemplify this by synthesizing glycerol trilevulinate (GT) from levulinic acid and glycerol wastes. The authors tested GT concentrations between 2.5 and 10 wt% in polyhydroxybutyrate (PHB), observing significant results at 5 wt%. These can be attributed to a 10 °C reduction in *T*_g_ and a 28% decrease in viscosity.

These changes enabled 3D printing at 180 °C without thermal degradation. Pure PHB requires 200 °C to obtain well-defined pores. In contrast, PHB with 5 wt% GT prints regular filaments at only 180 °C. In contrast, commercial plasticizers (acetyl tributyl citrate, ATBC or diisononyl cyclohexane-1,2-dicarboxylate, DINCH) performed poorly at this same temperature. Formulations with 5 wt% of ATBC or DINCH showed local filament irregularities and loss of pore geometry. This indicates that commercial options provide inferior melt control compared to GT. In a related study, Ghosh et al. [29] valued spent coffee grounds to produce coffee oil epoxide (COE). This biobased plasticizer was applied as a dual-function agent in poly(hydroxybutyrate-co-hydroxyvalerate) (PHBV)/Natural Rubber (NR) blends. Even at a low concentration of 0.3 wt%, COE significantly altered the mechanical profile, reducing the flexural modulus by 36.8%. Additionally, the material’s aesthetics improved, evidenced by a 7.73% increase in lightness. Beyond plasticization, the epoxide groups acted as interfacial compatibilizers; their chemical interactions transformed the inherently immiscible PHBV/NR blend into a single-relaxation system. This structural refinement was clearly confirmed via Dynamic Mechanical Analysis (DMA). The most striking results, however, were observed in the barrier properties. The improved interfacial adhesion led to a 98.9% reduction in oxygen permeability (OTR). Similarly, the water vapor barrier properties improved by 61.7%. These findings demonstrate that COE not only softens the matrix but also creates a more tortuous path for gas molecules.

Expanding on this strategy, Vallin et al. [30] utilized cashew nutshell liquid (CNSL) to develop a cardanol-derived diol, which was reacted with bio-succinic or adipic acids to produce bio-polyesters. These additives were synthesized via solvent-free enzymatic polycondensation using Lipase B and biomass-derived MeTHF at temperatures below 90 °C, effectively eliminating fossil-based components from the lifecycle. When incorporated into PLA (2–10 wt%), these bio-polyesters significantly improved flexibility; specifically, the PLA/DMA10 formulation reached 34% elongation at break. Notably, the stiffening effect was minimized (modulus remained stable at approximately 1872 MPa) due to the internal lubrication provided by the long aliphatic chains of the cardanol derivatives.

The development of high-performance bioplastics faces a critical trade-off between immediate plasticization efficiency and long-term additive permanence. To address the migration vulnerability of low molecular weight (LMW) plasticizers, De Bruyne et al. [31] synthesized citric acid-derived Tricarballylic acid (PTA) esters, specifically tributyl propane-1,2,3-tricarboxylate (TBPTC). At a content of just 5 wt%, TBPTC reduced the *T*_g_ of PLA from 63 °C to 38 °C while maintaining aqueous leaching below 1%. However, despite strong chemical affinity validated by radial distribution function (RDF) molecular dynamics, these LMW additives remain susceptible to significant volatilization during industrial processing at 200 °C. Alternatively, Montes et al. [32] mitigated volatility and migration risks using a polymeric plasticizer, poly(diethylene glycol adipate) (*M*_n_ = 2500 g/mol). Although this strategy requires higher loadings (10–15 wt%) in PLA/PHB blends, it yields superior ductility for food contact applications. A PLA/PHB 80/20 blend with 10 wt% of the polymeric additive achieved a 57.5% strain at break, significantly exceeding the 20–26% elongation range typical of LMW tricarboxylates.

Furthermore, these approaches diverge in their secondary functionalities. While PTA esters offer tunable flexibility via alkyl chain modification, the polymeric plasticizer prioritizes phase compatibilization, refining blend morphology, reducing porosity, and providing an effective UV barrier (T < 1% at 220–380 nm). The polymeric route also ensures a robust end-of-life profile, promoting over 90% disintegration under composting conditions within 35 days via accelerated hydrolysis. Ultimately, LMW esters maximize plasticization efficiency (“power per gram”), whereas polymeric additives deliver the thermal resilience and long-lasting miscibility essential for scalable sustainable packaging [31,32].

Chemical biomimicry in bioplastic engineering transcends the simple use of renewable monomers by emulating nature’s structural logic to create self-compatible macromolecules. For instance, Palenzuela et al. [33] replicated the cyclic multifunctionality of natural terpenes by synthesizing polylimonene oxide (PLO) via room-temperature aluminum-catalyzed ring-opening polymerization (ROP). When melt-blended into PLA at 5 to 20 wt%, PLO promoted partial miscibility via hydrogen bonding, significantly reducing the *T*_g_ (from 57 °C to 50 °C), crystallinity (from 13 to 1%), and flexural modulus (from 0.8 to 0.41 GPa). Crucially, PLO maintained terpene-like cyclic rigidity while optimizing industrial thermal processing, showing less than 4% weight loss at 180 °C. Alijanian et al. [34] mimicked the triglyceride architecture of vegetable oils by designing tri-armed bioresins (S3 and S7) from glycerol and oligo(lactic acid). Solution-casted into PLA films at 10–20 wt%, these lipid-mimetic architectures significantly enhanced mechanical toughness, increasing elongation at break from <20% (neat PLA) to approximately 150% for the 20 wt% S3 formulation. This transition from brittle to ductile behavior was accompanied by improved water vapor barrier properties; the bioresins acted as nucleating agents, increasing crystallinity and creating a more tortuous path for water molecules. However, the S3 formulation exhibited slightly reduced *T*_g_ and higher ethanol migration compared to its longer-armed S7 counterpart.

While PLO excels in thermal stability for melt processing, the triglyceridic bioresins still require refinement in green metrics for true scalability (EcoScale 75; PMI 2 kg/kg). Collectively, both paradigms highlight a shift toward endogenous, chemically homologous additives. By resolving brittleness through durable intramolecular interactions rather than passive blending, these bioinspired approaches pave the way for next-generation bioplastics in packaging and additive manufacturing [33,34]. A summary of the synthesis routes, polymer matrices, and experimental dosages for these and other recent bio-based plasticizers is provided in Table 2.

The search for bio-based plasticizers is no longer limited to simply “softening” the polymer but has evolved toward molecules that combine flexibility and flame safety within a single structure. Sun et al. [38] exemplify this trend with the development of phosphaphenanthrene groups-containing maleic acid 2-(2-n-butoxyethoxy) ethanol lactate ester (PMBL), whose synthesis route starts from corn to obtain L-lactic acid, which is then extended with ethoxyethanol chains and functionalized with maleic anhydride to enable final anchoring of the 9,10-dihydro-9,10-oxa-10-phosphaphenanthrene-10-oxide (DOPO) group. This molecular architecture not only reduces the rigidity of Polyvinyl Chloride (PVC) but also employs the phosphorus-containing unit to form a protective char layer, making the material simultaneously more flexible and less flammable. Similarly, Hou et al. [36] propose Phosphorylated Crotonic acid Lactate ester (PBCL), a lactic oligomer for PLA. Lactate “backbone” structure is optimized to lower the glass-transition temperature, while the phosphorus density is tuned via DOPO incorporation to promote stable char formation and reduce heat release during combustion tests. In contrast, Qian et al. [37] take an aromatic pathway starting from vanillic acid to develop Vanillic acid-based phosphate ester (VA8-P). This is a structure centered on the vanillic ring, where the addition of phosphate units and lateral alkyl chains enhances PVC flexibility and migration resistance while also significantly improving thermal stability. This integrated molecular design replaces the traditional use of separate additives, combining renewable flexible segments with flame-retardant groups to eliminate toxic plasticizers and halogenated flame retardants in simpler formulations.

The convergence of these studies also lies in their functional efficiency at low dosages, avoiding additive overload that could compromise processing or industrial cost. While Sun et al. [38] demonstrate that only 28.6 wt% (40 phr) of PMBL outperforms conventional ATBC in both flexibility (elongation of 648% vs. 395%) and flame retardancy (peak heat release rate (pHRR) reduced by 61%, char residue 12.5%), Hou et al. [36] optimize PBCL in PLA at just 16.7 wt% (20 phr) with a DOPO:BCL ratio of 0.2:1, achieving UL-94 V-0 classification and 434% flexibility without loss of transparency, a modest loading that maintains a low *T*_g_ (−25 °C) without the excessive stiffness observed at higher levels. Qian et al. [37], on the other hand, scale VA8 P to 37.5 wt% (60 phr) in PVC, reaching a limiting oxygen index (LOI) of 37% and elongation of 1142% (vs. dioctyl phthalate (DOP)), while reducing migration by 50%. However, they point out that intermediate dosages (16.7–28.6 wt%) already balance plasticization and char formation (20.5% residue), questioning the necessity of higher concentrations in petrochemical matrices.

The dual functionality of these bio-based additives lies in the precise balance between physical-chemical mechanisms. For plasticization, the aliphatic segments and ester groups, such as the ethylhexyl chains in VA8-P [37] or the lactate oligomers in PBCL [36] and PMBL [38], interleave between polymer chains to disrupt intermolecular forces. This process increases the matrix’s free volume and reduces the glass transition temperature (*T*_g_), enabling greater macro-scale flexibility. Concurrently, the flame-retardant mechanism operates through a combination of condensed and gas-phase actions. The phosphorus-containing moieties within these hybrid molecules, such as DOPO or phosphate esters, catalyze the formation of a stable, carbonaceous char layer during thermal degradation. This char acts as a physical barrier against heat and oxygen, while the potential release of phosphorus radicals helps quench combustion reactions.

The pursuit of multifunctional additives extends beyond mere combinations of plasticizers and flame retardants, and unfortunately, conventional literature often addresses plasticization and functionalization as distinct processes or reliant on complex mixtures of components. The development of Triphenyl Acetic Glyceroate (TPAG) by Ferri et al. [35] consolidates the “all-in-one-molecule” concept, synergistically combining plasticizing effects with gas barrier properties, antioxidant activity, antimicrobial action, and UV blocking, thereby rendering PLA viable for fresh food preservation (tested on pear slices). Unlike traditional biobased additives, such as citrates or epoxidized vegetable oils, which primarily target reductions in glass transition temperature (*T*_g_), TPAG acts synergistically as both a barrier agent and oxidative stabilizer. Quantitative analysis of TPAG incorporation reveals a critical dose-dependency for the transition from a glassy to a flexible state, wherein increasing from 0 to 16.7 wt% results in a linear *T*_g_ reduction from 57 °C to 35 °C. This plasticizing effect is accompanied by a dramatic shift in mechanical properties: while neat PLA exhibits only 11% elongation at break, the 16.7 wt% formulation achieves 599%. Regarding barrier functionality, TPAG introduces a competitive edge over low-density polyethylene (LDPE) by reducing water vapor permeability (WVP) with increasing concentration, a phenomenon attributed to the steric hindrance of aromatic rings. Additionally, active protection is dose-scaled, reaching 94% UV-C blocking at the maximum concentration. However, this study establishes a crucial regulatory safety threshold: overall migration in dry food simulant (Tenax^®^) remains below the European limit of 10 mg·dm^−2^ only up to 9.1 wt% (10 phr), underscoring that while 16.7 wt% optimizes ductility, food contact viability peaks at the intermediate content from both technical and normative standpoints.

The evidence suggests that the synergy between condensed-phase mechanisms (char formation) and gas-phase inhibition as seen in urushiol and DNA-functionalized lignin is the most viable route to achieve UL-94 V-0 ratings without the mechanical trade-offs typical of traditional halogen-free systems. Furthermore, the development of bio-vitrimers and covalent grafting strategies (notably LNC-g-PLA) points toward a future where fire safety is an intrinsic property of the polymer architecture rather than a secondary modification. For future research, the challenge lies in balancing these high-performance chemical modifications with green metrics, ensuring that the synthesis of these bio-additives remains solvent-free and scalable for industrial adoption. Ultimately, these advances provide a strategic roadmap for engineering a new generation of polymers that are not only fire-safe but also fully compatible with the circular economy and biological end-of-life pathways.

#### 3.2.2. Sustainable Flame Retardants

**Figure 4:**
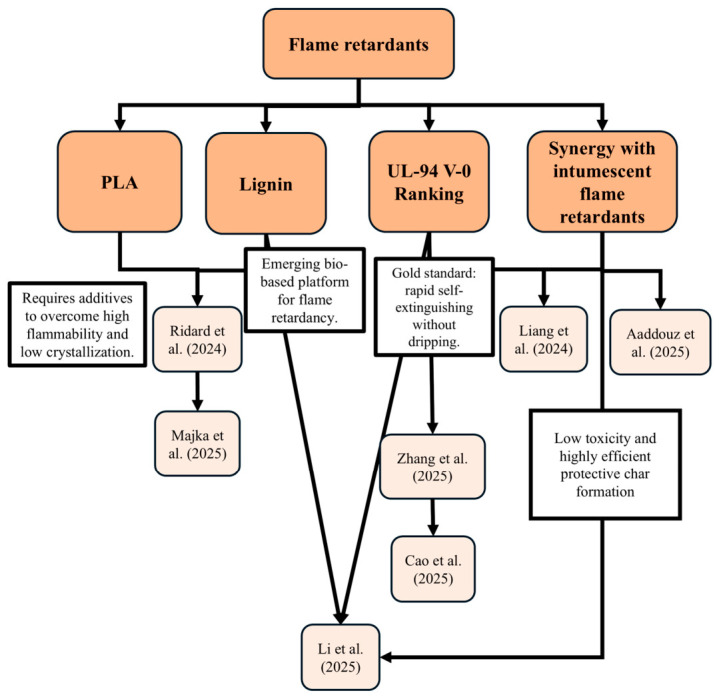
Principal approaches and key authors in the development of sustainable flame retardants [26,39,40,41,42,43,44].

The development of sustainable flame retardants has converged toward the valorization of lignin as a versatile platform, transcending its traditional role as a mere filler to serve as a precursor for multifunctional additives. This trend, synthesized in Figure 4, reflects a transition from physical blending toward macromolecular engineering and biological functionalization.

Li et al. [39], and Majka et al. [40] reveals a robust trend in sustainable materials development, positioning lignin as a key component for flame retardancy. The former group explores lignin functionalization with Deoxyribonucleic acid (DNA) to create biological additives, while the second study advances toward vitrimers where the polymer structure itself is primarily composed of epoxidized lignosulfonate, achieving intrinsic fire resistance. In parallel, research by the latter investigates the potential of modified lignosulfonated bio-fillers, such as those treated with tannic and gallic acids to enhance the thermal performance of polymer blends.

The lignin concentrations employed across these studies reflect distinct optimization strategies. For instance, incorporating up to 20 wt% of functionalized nanoparticles enabled the development of lightweight, nucleated PLA foams, although it led to a decrease in initial thermal stability and polymer chain length [26]. In a different approach, a 3 wt% loading of bio-fillers in polymer blends proved beneficial for maintaining overall thermal stability; this concentration was sufficient for gallic acid-modified additives to achieve an FV-1 rating, whereas it remained inadequate for less reactive derivatives such as calcium lignosulfonate [40]. Conversely, employing high proportions, up to 85 wt% of lignosulfonate in vitrimer formulation ensured superior intrinsic flame retardancy and a UL-94 V-0 rating. This method leveraged lignin’s aromatic compounds to form dense char layers, though the inherent brittleness caused by such high concentrations required compensation with bisphenol A diglycidyl ether (DGEBA) to maintain processability and material homogeneity [39].

A common finding across all three studies is lignin’s ability to promote the formation of a stable carbonized layer, known as char. In the DNA-functionalized system, the synergy between phosphorus and the lignin framework effectively reduced the total heat release, validating the efficiency of this biological approach. This is due to DNA’s intrinsic intumescent character, where phosphate groups act as acid sources and nitrogenous bases release non-combustible gases, promoting lignin carbonization [26]. However, a shared challenge particularly noted in the investigations involving PLA matrices was the reduction in initial thermal stability and accelerated ignition in specific scenarios. This suggests that while these additives provide superior protection during advanced burning stages, they may trigger earlier degradation [26,40].

The technical limitations associated with poly(lactic acid) matrices are further evidenced by processing difficulties, specifically regarding matrix immiscibility and molecular weight reduction. The latter is often driven by the interaction of lignin’s phenolic and carboxylic groups with the polyester chains, while in more complex systems, such as PLA/PET blends, the lack of compatibility is clearly manifested by distinct glass transition temperatures [40]. Beyond flame retardancy, lignin’s multifunctional nature allows it to act as an efficient nucleating agent. This property has been successfully leveraged to develop lightweight PLA foams with refined cell morphology [26], as well as to modify the crystallization behavior of the matrix using derivatives like calcium lignosulfonate (CLS) [40]. However, the performance is highly derivative-specific; for instance, while modified fillers show promise, untreated CLS or lignosulfonamide (SA) often fail to meet stringent fire safety standards like the V-0 rating. Consequently, surface-level engineering, such as grafting PLA chains onto lignin nanoparticles (LNC-g-PLA), emerges as a superior strategy to ensure interfacial adhesion and homogeneous dispersion within the biopolymer [26].

A synthesis of recent studies underscores a strategic shift toward achieving the maximum fire safety standard, the UL-94 V-0 rating, across diverse polymer systems. Zang et al. [41] met this objective by incorporating 10 wt% of a phytic acid phenethylamine (PA1PEA12) into polyhydroxyalkanoate (PHA) matrices, which eliminated secondary ignition risks from dripping and increased the Limiting Oxygen Index (LOI) to 29.7%. This performance is complemented by the findings of Liang et al. [42], who utilized 9 wt% of a phosphonate derived from urushiol resin (U-DC) to convert epoxy resin into a V-0 material, reaching a remarkably high LOI of 37.1%.

Notably, these high-safety classifications were achieved without compromising structural integrity. While the urushiol-based additive reinforced the mechanical strength of the epoxy, the phytate derivative preserved over 90% of the PHA’s original impact resistance. This balance between safety and performance finds a robust parallel in the work of Li et al. [39], who obtained intrinsic UL-94 V-0 ratings using high concentrations (up to 85 wt%) of epoxidized lignosulfonate. Unlike traditional additive strategies, this system leverages lignin’s inherent aromatic network to suppress melt-dripping, by increasing melt viscosity and promoting rapid cross-linking during heating, which stabilizes the polymer melt. Similarly, Cao et al. [43] demonstrated that incorporating 11 wt% protonated chitosan (PCS) into PVA films achieved not only a V-0 rating but also a significant reduction in heat release rates, surpassing mitigation indices observed in other contemporary systems. The efficiency of these studies regarding the V-0 standard is predominantly based on condensed-phase mechanisms, where the formation of a char barrier plays a central role. In both the PHA composites and lignin vitrimers, the carbon layer acts as a physical barrier against oxygen and heat, a process also observed in urushiol-modified epoxy resin, which forms a dense, crack-free film. For the chitosan-PVA system, this carbonization is catalyzed by the additive, where acidic species released from the phosphorous acid-protonated chitosan promote the dehydration and dehydroxylation of the PVA matrix. This leads to early char formation and a 10-fold increase in residue mass compared to neat PVA. Collectively, these results demonstrate that concentrations ranging from 9 to 11 wt% for specific additives, or the predominant use of functional biopolymers as seen with lignin, represent scalable routes to produce materials that balance circular economy goals with the highest technical fire safety requirements.

Beyond conventional systems, the search for synergy in intumescent protection has led to the exploration of polyphenolic platforms such as tannic acid and urushiol. Recent research highlights how transitioning to these renewable sources ensures efficient matrix carbonization [39,42,44]. Specifically, the work of Aaddouz et al. [44] demonstrated that modifying tannic acid with potassium phosphate (AT-PK) at a 30 wt% loading results in a 78% reduction in the pHRR. This performance is primarily attributed to the formation of an expanded intumescent char layer that acts as a physical barrier against heat and mass transfer. However, despite achieving high dispersion, this tannic-based system exhibited low initial thermal stability, which reduced ignition time and failed to prevent dripping in UL-94 tests. This indicates that while effective in reducing fire intensity, synergy with secondary additives remains essential for meeting stringent vertical safety ratings [44].

In contrast, the urushiol-derived phosphonates (U-DC), as previously reported, demonstrate a superior dual-phase mechanism: releasing phosphorus radicals in the gas phase while promoting a dense, crack-free char layer in the solid phase. Such high solid-phase efficiency not only suppresses flame spread but also enhances the mechanical strength and flexibility of the resin, addressing the common brittleness of high-filler systems [42]. Complementing these additive-based strategies, as previously discussed, sustainable vitrimers offers a definitive pathway toward intrinsic flame retardancy. By integrating biological precursors directly into the polymer network, immediate self-extinction is achieved through a condensation mechanism that forms an extremely dense ash layer [39]. While studies on urushiol and tannic acid focus on the functionalization of pre-existing matrices [42,44], this structural integration of lignin-based precursors provides superior thermal stability and solvent resistance, consolidating a self-sufficient carbon network that protects the underlying material [39].

#### 3.2.3. Natural Antioxidants

Lignin has also been extensively investigated as a natural antioxidant in polymer matrices, revealing a performance duality highly dependent on botanical origin, isolation methods, and, fundamentally, the concentration utilized. Recent literature highlights a significant methodological contrast in lignin integration strategies. Pappa et al. [45] explored organosolv beech lignin (OBs) in epoxy resins at high loadings, ranging from 3 to 16.6 wt%. In contrast, Babetto et al. [46] investigated eucalyptus-derived lignins for the stabilization of recycled polypropylene (rPP) at significantly lower concentrations, between 0.05% and 0.5 wt%. Comparative analysis indicates that antioxidant efficiency can be superior at low dosages; specifically, the incorporation of only 0.2 wt% of certain lignin fractions can outperform commercial synthetic antioxidants in oxidation induction time (OIT) tests and stabilization of molecular weight [46]. In contrast, the maximum radical scavenging activity (specifically approximately 80% against DPPH) in epoxy systems required higher concentrations and prolonged reaction times (up to 96 h), suggesting that while OBs lignin acts as an efficient mechanical reinforcement at 3 wt%, increasing strength by up to 36%, excessive loadings near 16.6 wt% result in material embrittlement [45].

From a qualitative perspective, the density of phenolic hydroxyl groups remains the primary determinant of stabilization performance, and these groups act as primary antioxidants by donating hydrogen atoms to peroxy radicals, effectively terminating the radical chain reaction during polymer degradation. [45,46]. And lignin fractions rich in sterically hindered guaiacyl and syringyl units provide superior thermal protection under severe processing conditions (240 °C), maintaining polymer viscosity more effectively than conventional additives. This is attributed to the methoxy groups in the ortho-positions that stabilize resulting phenoxyl radicals via electronic resonance and inductive effects, effectively inhibiting the thermo-oxidative degradation chain [40]. Furthermore, the versatility of these macromolecules allows them to function simultaneously as antioxidants and curing agents, improving the Young’s modulus of resins by 12–16%. However, distinct challenges persist depending on the dosage and extraction pathway [45]. High-loading strategies often face a critical loss of ductility, whereas low-concentration stabilization is heavily influenced by the extraction pH, as only fractions obtained under more acidic conditions exhibit the necessary purity for effective stabilization [45,46].

Expanding the analysis to biodegradable polymers, the use of agro-industrial by-products and green lignin derivatives has revealed distinct functional stabilization strategies for matrices such as poly(ε-caprolactone) (PCL) and poly(butylene succinate) (PBS). Melro et al. [47] focused on lignin extracted via levulinic acid (LA-lignin) and other commercial variants within a PCL matrix. In a different approach, Hiller et al. [48] investigated the direct incorporation of wine pomace (WP) as a functional filler in PBS.

Regarding the concentrations utilized, a discrepancy in efficiency was observed between the two systems. Specifically, a high radical scavenging activity (RSA) of 91.7% was achieved with only 5 wt% of lignin, reaching a plateau of 95% at higher loadings [47]. Conversely, low dosages of WP, specifically 3 wt%, were found to be sufficient to increase the thermo-oxidative stability of PBS by at least 24%. This establishes the residue as an efficient stabilizer at minimal concentrations without altering the melting and crystallization behavior of the matrix [48].

Qualitative performance comparisons indicate that LA-lignin yields the best overall results in terms of bioactivity, with an RSA of 94.8%, outperforming alkaline lignins and maintaining thermal preservation levels close to the neat matrix, which suggests superior compatibility [47]. On the other hand, the use of WP offers significant industrial reliability, as findings prove that seasonal variations and grape varieties do not compromise the thermal properties of the biocomposite, maintaining a stable melting temperature (*T*_m_) even at 20 wt% loading [48]. However, both studies report a negative impact on mechanical properties and stability at high concentrations. A drastic drop in tensile strength, from 21 MPa to 9 MPa at 50 wt% lignin, was reported due to poor interfacial adhesion [47]. Similarly, it was observed that WP contents above 5 wt% begin to moderately reduce the intrinsic thermal stability of the system [48]. These findings underscore that while these bio-additives are highly effective for oxidative protection, their dosage must be carefully optimized to preserve the structural integrity of the biopolymer [47,48].

The optimization of poly(3-hydroxybutyrate-co-3-hydroxyvalerate) (PHBV) performance using food waste derivatives has proven to be an effective strategy for both enhancing thermal stability during industrial processing and providing advanced functionality for packaging. Rusko et al. [49] and Ferri et al. [50] converge in identifying concentrations near 5 wt% as the ideal equilibrium point for biocomposite performance. While the former focused on oxidative stability during continuous extrusion, utilizing aronia pomace and lima bean husks to prevent viscosity drop and maintain chemical integrity up to 200 °C, the latter explored the use of tannins via solvent casting to confer multifunctional properties. Regarding stabilization efficiency, both approaches successfully maintained thermal stability but achieved distinct functional outcomes. The incorporation of 5 wt% lima bean husks outperformed petrochemical antioxidants in preserving melt viscosity under shear conditions [49]. In contrast, a PHBV formulation containing 4.76 wt% tannin (PHBV-tan5) stood out for its superior gas barrier properties (CO_2_ and O_2_) and its capacity for colorimetric detection of food spoilage, mediated by the structural sensitivity of tannins to alkaline environments and volatile amines, triggering a bathochromic shift (darkening) as a visual response to metabolic by-products [50].

From a technical perspective, the validation of large-scale continuous manufacturing viability is a significant advantage, proving that regenerative additives can replace fossil-based antioxidants without altering the polymer’s molecular structure [49]. Furthermore, the integration of tannins adds multifunctionality by providing UV protection and intelligent sensing capabilities to the material [50]. However, specific limitations persist in both systems. Residual moisture in natural additives may cause minor mass losses during processing, and mechanical properties remain a secondary focus in certain extrusion-based studies [49]. Regarding tannin-rich formulations, increasing the concentration to 10 wt% results in excessive loss of transparency and elevated stiffness evidenced by a 20% increase in Young’s modulus which may restrict specific packaging applications [50]. Collectively, these studies confirm that bio-based additives in the 5 wt% range significantly enhance PHBV, with lima bean husks being more suitable for thermal processing resistance and tannins for active and intelligent packaging systems [49,50].

Given the diversity of sources, matrices, and methodologies analyzed, the selection of a natural antioxidant transcends simple sustainable replacement, representing a strategic decision to confer specific properties to the final material. To facilitate comparison between the different technological approaches and their impacts, Table 3 synthesizes the main differentiators and concentrations investigated in the discussed studies, highlighting the innovations that each residue provides to polymer science.

The data integrated from recent literature establish a technical basis for optimizing biopolymer formulations through renewable stabilization. Evidence indicates that the successful application of natural antioxidants depends on a precise balance between chemical purity and dosage. While ultra-low concentrations (0.05–0.5 wt%) of highly phenolic lignins are sufficient for commodity polymers like recycled PP, complex functionalities, such as active sensing in smart packaging or reactive curing in thermosets, require higher loadings and specific structural modifications.

The mapping of these research fronts highlights the necessity of correlating extraction methods with final stabilization efficiency. These findings allow for the strategic selection of biomass precursors based on industrial processing temperatures and the required end-of-life profile, reducing the need for empirical trials. Ultimately, the transition to multifunctional bio-additives mitigates polymer degradation while advancing the development of high-performance materials aligned with circular economy frameworks.

#### 3.2.4. Compatibilizers

The evolution of interfacial agents reflects a strategic transition from reliance on fossil-based synthetic polymers to the use of renewable precursors with high reactive performance. While early studies were largely centered on petrochemical-derived graft copolymers, contemporary research, such as that by Srisuwanno et al. [51] and Morinval and Avérous [52], emphasizes the effectiveness of functionalized elastomers and finely tuned chemical modifications. Specifically, the use of epoxidized natural rubber (ENR) and the development of macromolecular architectures via thiol–Michael “click” reactions illustrate that modern compatibilization is no longer limited to reducing interfacial tension. Instead, these approaches promote the formation of covalent chemical bonds that refine phase morphology and ensure robust interfacial adhesion, enabling the creation of multiphase systems with properties once exclusive to conventional engineering polymers.

The effectiveness of these reactive strategies is evidenced by the sensitivity of mechanical properties to the compatibilizer content and structure. In the thermoplastic elastomer-based system, the incorporation of only 2.9 wt% of epoxidized natural rubber (ENR) into TPU/NR blends increased the tensile strength by 41.1% and the elongation at break to 278%, indicating that small amounts of such bio-based modifiers can drastically enhance load transfer. This is facilitated by the chemical interactions between the oxirane/hydroxyl groups of the ENR and the polar urethane segments of the TPU, leading to improved interfacial adhesion and the formation of a stable phase-bridging structure [51]. Complementarily, Morinval and Avérous [52] synthesized complex macromolecular architectures via base-catalyzed thiol–Michael click addition between maleimide-functionalized amylomaize starch (AMS) and thiolated PBAT oligomers. This fast and selective mechanism creates a cross-linked hybrid network that effectively bridges the gap between the hydrophilic polysaccharide and the hydrophobic copolyester. The resulting insoluble covalent architecture demonstrates that such ‘click’ linkages provide high grafting density at the interface, which is important for stabilizing the morphology and preventing phase coalescence in starch/PBAT blends for high-performance applications. While this study serves as a successful proof of concept for the synthesis of these architectures, their practical performance as compatibilizers in melt-processed blends remains to be further evaluated.

In parallel with the development of macromolecular architectures, authors such as, Arslan et al. [53] and Jing et al. [54] have directed their efforts toward overcoming the inherent brittleness of poly(lactic acid) (PLA) through the use of toughening agents. The central challenge lies in achieving increased ductility without compromising the characteristic stiffness of the matrix, a technical barrier that both studies seek to overcome by compatibilizing hybrid systems. While the former explores the synergy between bio-based elastomers and mineral reinforcements (basalt fibers), the latter investigates the potential of additives with dual functionalities, plasticizing and reactive compatibilization, in PLA/PBAT blends. This joint approach reinforces that effective PLA toughening depends not only on the presence of a dispersed elastomeric phase but also on the compatibilizer’s ability to ensure that this phase is finely distributed and chemically anchored to the matrix.

Experimental data analysis reveals that the efficiency of these modifiers is highly dependent on the processing technique and the critical additive loading. In basalt fiber-reinforced biocomposites, incorporating 15 wt% of a bio-based hydrogenated styrenic thermoplastic elastomer (SEPTON) led to a remarkable 160% increase in impact strength (31.4 kJ/m^2^) and a 31% improvement in tensile strength (65.8 MPa) when processed by injection molding [53]. Complementarily, the investigation of binary bio-blends demonstrated that the introduction of 10.7 wt% (12 phr) of a multifunctional reactive agent (DBI) resulted in a 13.81 °C decrease in *T*_g_ and increase in elongation at break, reaching 620.6%, a value five times higher than that of the neat system. This phenomenon is attributed to DBI that acts as a reactive bridge, under the influence of the radical initiator (DCP), its unsaturated C=C double bonds facilitate chemical grafting onto both PLA and PBAT backbones. This interfacial reaction promotes covalent anchoring and reduces interfacial tension, transforming the morphology from a coarse ‘sea-island’ structure to a highly integrated co-continuous phase, which effectively toughens the matrix [54]. However, a common saturation limit was observed across these distinct strategies; the study on reactive plasticization reported that contents above 10.7 wt%, such as 12.3 wt%, lead to a decline in mechanical properties. This trend indicates that strict control of additive concentration is crucial to prevent phase separation and maintain the structural integrity of the eco-composite, regardless of whether the reinforcement is fibrous or elastomeric.

The shift from passive physical blending to precise interfacial engineering establishes a new benchmark for the development of high-performance bioplastics. The evidence across distinct polymeric systems suggests that structural optimization depends less on volumetric additive loading and more on the establishment of a specific ‘reactive window’, typically identified between 3 and 15 wt%, where covalent anchoring and phase dispersion reach a thermodynamic equilibrium. Beyond mere toughening, the integration of multifunctional bio-agents that combine plasticization with chemical coupling emerges as the most viable pathway to resolve the long-standing trade-off between stiffness and ductility in biopolymers such as PLA. Therefore, future progress in the field necessitates a move toward molecular designs that prioritize interfacial selectivity and thermal stability, ensuring that sustainable multiphase materials can meet the rigorous mechanical demands of industrial applications without compromising their circular end-of-life profile.

#### 3.2.5. Bio-Based Fillers and Reinforcements

Recent studies on additive manufacturing of PLA-based composites have focused on surface modifications and higher loading strategies to overcome intrinsic limitations such as brittleness and low thermal resistance. While Wu et al. [55] explored the structural potential of continuous flax fibers, Frasca et al. [56] and Ilhan et al. [57] focused on polymer matrix modification using lignin, polycaprolactone (PCL), and cellulose nanocrystals (CNCs), whereas Mandala et al. [58] investigated the technical and economic feasibility of employing carbon nanoparticles derived from agricultural residues.

The efficiency of mechanical reinforcement in PLA is strongly influenced by the morphology and orientation of the filler, as demonstrated by Wu et al. [55], who achieved a 2.6-fold increase in tensile strength and a 3.8-fold increase in Young’s modulus by aligning continuous flax fibers at 0° relative to the loading direction. However, this performance gain is accompanied by pronounced anisotropy, as 90° orientations led to strength reductions of up to 85% due to the predominance of interlayer adhesion over fiber strength. At 90°, the load is primarily sustained by the weaker polymer-fiber interface and the bond between printed layers, leading to premature transverse failure. This interfacial dependence is also a challenge reported by Frasca et al. [56], who employed high lignin loadings of 30%, 50%, and 70% by weight. Although phenolated organosolv lignin (POL) at 30% maintained stable mechanical properties and excellent printability, 70% loadings caused drastic strength reductions due to filler agglomeration and the inherently lower mechanical strength of the biomass.

The toughening strategy of PLA, aimed at reducing its brittle nature, was further explored by Ilhan et al. [57], who proposed a PLA/PCL blend reinforced with 5 wt% cellulose nanocrystals (CNCs). In contrast to continuous fiber reinforcements that primarily target stiffness, the addition of 20 wt% PCL transformed the material behavior from brittle to ductile, increasing the elongation at break from 6.76% to 40.25%. However, this gain in ductility was accompanied by a decrease in elastic modulus. This reduction is likely attributable to the dominant softening effect of the PCL phase, which appears to have overshadowed the stiffening potential of the CNCs. This was further compounded by phase immiscibility and the reported CNC agglomeration, which likely hindered effective stress transfer and prevented the formation of a reinforcing network. A similar sensitivity to filler integration was observed by Frasca et al. [56], who noted that lignin, although sustainable, tends to make the composite more brittle under certain conditions.

In parallel with the development of complex filaments, Mandala et al. [58] introduced an alternative processing route by employing direct pellet extrusion and incorporating 0.25 wt% carbon nanoparticles (GNSC) synthesized from peanut shells. This simplified approach achieved a tensile strength of 51.744 MPa, a value that compares favorably with the POL30 blends studied by Frasca et al. [56] and the nanocomposites reported by Ilhan et al. [57], whose strength tended to decrease to the 37 to 44 MPa range upon the addition of PCL and CNCs. The effectiveness of these nanoparticles at extremely low concentrations contrasts sharply with the high filler loadings of lignin (up to 70%) or continuous fibers, suggesting that interfacial optimization and nanoscale dispersion may represent more efficient pathways to preserve the structural integrity of PLA without compromising its processability.

Within the body of research analyzed, a clear evolution in the scientific conception of lignin’s role in polymeric matrices is observed, shifting from a mere plant byproduct to a multifunctional additive capable of modulating structural and thermal properties. The study by Sanchez-Sobrado et al. [59] reinforces this perspective by employing high concentrations of nanolignins (15–35 wt%) in high-density polyethylene (HDPE), targeting industrial applications in high-stiffness composites. In contrast, Banpean et al. [60] demonstrate that lignin performance is not strictly contingent upon high filler loading, but rather on its morphology and chemical affinity with the matrix; they showed that a mere 1 wt% of spherical lignin nanoparticles (SLNs) was sufficient to induce heterogeneous nucleation in poly(L-lactic acid) (PLLA). This divergence illustrates that the function performed by lignin, whether as a structural reinforcement or as a crystalline morphology modifier is heavily dependent on interfacial interaction and the dimensional regime of the particles.

Sanchez-Sobrado et al. [59] identified that increasing the nanolignin content from 15 to 35 wt% yielded an approximate 60% enhancement in both the elastic modulus and flexural strength of HDPE. This behavior is attributed to the formation of a continuous internal network once the percolation threshold is reached between 30 and 40 wt%. However, this reinforcing trend was non-linear; concentrations exceeding this threshold resulted in a decline in tensile strength due to the formation of aggregates that act as stress concentration points. Such reinforcing limits are highly dependent on the polymer matrix; for instance, Frasca et al. [56] reported a decrease in tensile properties at loadings as low as 5 wt% in PLA, highlighting that the critical concentration for mechanical failure varies significantly across different systems. Contrasting data were reported by Banpean et al. [60], where the introduction of 1 wt% SLNs into PLLA reduced the crystalline induction time from 330 to 120 s and increased total crystallinity by up to 9% without compromising mechanical integrity, an indicator of effective dispersion and favorable surface compatibility. Thus, while the high stiffness achieved in the HDPE system stems from the restriction of chain mobility, the efficient nucleation process observed in PLLA derives from the intensification of the contact surface at low concentrations [59,60]. This reveals that final performance is not merely a function of the quantity added, but primarily of the nature of the interactions between the polymer and lignin.

The increasing incorporation of lignocellulosic fillers into bioplastic or recycled matrices reflects a paradigm shift in sustainable materials development, aiming to harmonize mechanical performance with circularity. Schulz et al. [61] explore this trend by demonstrating the effects of adding plant-based flours derived from residues such as wood and olive pits. On an upscaled level, the study by Romero-Ocaña et al. [62] advances this premise by focusing on the chemical compatibilization of ABS composites reinforced with cork particles. Their findings highlight that the primary challenge lies not merely in waste incorporation but in establishing stable interfaces between phases of contrasting polarities. In both studies, filler efficiency depends less on the total loading and more on the ability to modify the internal stress field of the polymer, acting as anchoring points that restrict chain mobility. Thus, functionalization and interfacial control strategies emerge as central elements in the transition from simple physical filling to an effective and technologically viable structural reinforcement system.

Schulz et al. [61] observed a significant increase in the tensile modulus, from 190 MPa to 593 MPa, with the addition of only 0.5 wt% of olive pit flour. This result underscores the dispersion efficiency and the mechanical nucleation role of finely distributed particles. In this instance, the stiffness enhancement stems not from the quantity but from the homogeneous spatial arrangement of particles that inhibit polymer chain flow under stress. In contrast, Romero-Ocaña et al. [62] employed a higher concentration of 10 wt% cork in ABS, achieving an elastic modulus of 1515 MPa and a tensile strength of 41.05 MPa. These values are comparable to those of the neat polymer, despite a moderate reduction in ductility. This performance is attributed to the synergistic use of maleic anhydride (MAH) and dicumyl peroxide (DCP) as compatibilizers, which facilitated covalent bonding between the ABS chains and the hydroxyl groups of the cork, thereby promoting interfacial strength and superior stress transfer. While the first system relies on the physical and morphological architecture of the particles, the second highlights the importance of surface chemical engineering, two complementary approaches that, when integrated, consolidate high-performance, renewable-origin bio-based composites.

While previous studies have emphasized dense matrices, a new frontier is emerging in the application of plant and algal residues within porous and recyclable systems. In these applications, lignocellulosic fillers transcend mere mechanical reinforcement to function as modifiers of processability and acoustic absorption. Rus et al. [63] exemplify this expansion by incorporating wood chips (5–20 wt%) into bio-epoxy foams derived from used cooking oil, revealing that the renewable origin of the matrix confers not only flexibility but also superior environmental resilience compared to synthetic epoxy. Complementing this approach, Matsumoto et al. [64] demonstrate the potential of agro-industrial by-products, such as citrus fiber, in biological polyester films. These fillers outperform expensive reinforcements like nanofibrillated cellulose due to their linear structure and dispersibility. Furthermore, Yeh et al. [65] innovate by using Chlorella sorokiniana microalgae to enable the recycling of cross-linked EVA foams, overcoming thermoset barriers through natural plasticization. These studies converge on the principle that interfacial functionalization, whether through colloidal dispersion, controlled cross-linking, or physical barriers transforms residues into active agents, promoting multifunctional materials with a reduced environmental footprint and enhanced adaptability to extended life cycles.

Rus et al. [63] reported that the bio-epoxy (BE) containing wood chips exhibited a glass transition temperature (*T*_g_) of 100 °C, which is lower than the 130 °C observed for synthetic epoxy (SE), reflecting shorter chains and inherent flexibility. However, after 6000 h of UV exposure, the BE maintained superior acoustic resilience, with a sound absorption coefficient (SAC) 12–18% higher at 3000 Hz, attributed to the natural antioxidants present in lignin. In addition to this environmental durability, Matsumoto et al. [64] demonstrated that the addition of 3 wt% citrus fiber to HP1 polyester doubled the tensile strength, from 6.41 MPa to 11.69 MPa, and increased toughness to 9.55 MPa. This performance surpassed dextrin and rivaled nanofibrillated cellulose due to homogeneous dispersion. Conversely, Yeh et al. [65] focused on EVA recycling, where microalgae concentrations of 23–33 wt% increased the melt flow index (MFI) from 2.5 g/10 min to 5–12 g/10 min, facilitating thermal reprocessing. These results highlight that bio-based fillers optimize specific properties, such as acoustics in BE, mechanics in films, and processability in EVA, while providing environmental benefits such as the sequestration of 1.83–1.88 kg CO_2_ per kg of biomass, reducing emissions by up to 63%.

#### 3.2.6. Others

The incorporation of bio-based additives allows for the precise modulation of mass transport, addressing the dual needs of selective permeation in membranes and impermeability in packaging. In filtration systems, Rezaei Khenari et al. [66] and Al Marri et al. [67] utilized additives like capsaicin, β-carotene, and carrageenan to modify PMP and PES membranes. By exploiting polar functional groups (such as sulfates and hydroxyls), these additives induce microstructural changes, potentially acting as pore-forming agents during phase separation, which is reported to increase surface hydrophilicity and mitigate foulant adhesion. Conversely, for packaging, Li et al. [68] and Yolacan and Deniz [69] focused on barrier performance using biowaxes and hydroxypropyl methylcellulose (HPMC). These materials utilize hydrophobic lamellae and hydrocolloid networks to create tortuous paths, effectively blocking water vapor and grease. This dichotomy highlights how the specific polarity of renewable additives can be tuned to either facilitate flow or suppress diffusion.

Experimental data confirms that dosage optimization is key to these functional shifts. In gas separation, 5 wt% capsaicin increased CO_2_ permeability in PMP by 53% (reaching 148 Barrer) while improving selectivity from 8.41 to 12.26 [66]. Similarly, a mere 0.5 wt% carrageenan loading in PES membranes tripled water flux to 1429 LMH and reduced fouling by 38% [67]. On the barrier spectrum, Li et al. [68] achieved maximum grease repellency (KIT 12) and water resistance (Cobb 14 g/m^2^) with 7.5 wt% biowaxes. Furthermore, HPMC-based blends significantly outperformed polyethylene references, reducing OTR to 14 cc/m^2^·day and WVTR to 9 g/m^2^·day [69]. Collectively, these studies show that while low loadings (≤5 wt%) are ideal for enhancing membrane flux, targeted concentrations are essential to establish robust barriers without compromising mechanical integrity.

Emerging bio-based additives address structural limitations by precisely controlling crystallization kinetics and packing architectures. Marchi et al. [70] employ a “molecular engineering” strategy, synthesizing renewable bis-α-ketoamides to nucleate PLLA through specific supramolecular interactions, where bis-α-ketoamides are suggested to self-assemble into nanofibrillar structures, providing epitaxial sites that may facilitate lamellar growth. In contrast, Carrero et al. [71] utilize the physical acicular morphology of sepiolite to modify the meso- and microstructure of bio-based PA11. Rather than altering chain chemistry, this approach reorganizes crystal packing and reduces anisotropy in FFF-printed parts. Thus, the field is advancing through two distinct pathways: “tailor-made” chemical signatures for kinetic tuning versus geometric exploitation of fillers for morphological control.

At a low loading of 1 wt%, Marchi et al. [70] increased the peak crystallization temperature (Tc) of PLLA by 5–7 °C. Although this shift is lower than commercial references (>10 °C), it represents a significant thermodynamic gain for a fully renewable system. Conversely, at higher loadings (5–15 wt%), Carrero et al. [71] demonstrated that sepiolite doubled the Young’s Modulus in the XZ orientation and increased it by 45% in the critical interlayer (YZ) direction. This performance, which surpasses equivalent glass fiber composites, stems from superior load transfer and microstructural refinement. Microtomography revealed that sepiolite induced an α-to-γ phase transition and progressively reduced pore diameter, creating a more isotropic structure unlike the irregular defects found in glass fiber systems. Ultimately, these findings confirm that optimizing sustainable polymers requires balancing low-dosage chemical nucleation for kinetic control with moderate-dosage physical fillers for structural reinforcement.

Surface engineering provides essential environmental protection for sustainable polymers through two distinct mechanisms: migratory self-healing and covalent anchoring. Peng et al. [72] utilized the amphiphilic nature of a cardanol-derived agent (BCP) to create a spontaneous lubricating “skin” on polypropylene, mimicking plant wax secretions. This behavior is likely driven by the limited thermodynamic miscibility of the additive, which promotes its migration toward the surface to form a low-energy layer. In contrast, Yang et al. [73] and Gaddam et al. [74] engineered intrinsic barriers using furan dioxime (DFFD) and maleinized cottonseed oil (MACSO). These covalent approaches avoid depletion issues, ensuring durable antimicrobial and anticorrosive performance via metal coordination and dense amide networks reinforced by graphenamine.

Experimental data reveals a convergence on a 3 wt% optimization threshold across these strategies. In the migratory system, 3 wt% BCP reduced scratch depth by 46% and color variation by 84% [72]. For covalent systems, optimized dosages of DFFD achieved 100% bacterial inhibition and enhanced adhesion strength to 3.85 MPa [73], while 3 wt% graphenamine in polyamide reduced the corrosion rate to 5.99 × 10^−6^ mm/year [74]. These findings suggest that regardless of the mechanism whether exploiting surface mobility or fixed chemical stability, this specific concentration represents the ideal balance for multifunctional protection.

In addition to structural enhancement, biomass plays a critical rheological role as a dispersing agent, acting as a facilitator for high-performance materials. Lage-Rivera et al. [75] illustrate this function by incorporating lignin and coffee grounds (CGG) into PLA composites reinforced with carbon nanotubes (MWCNT). Instead of acting as mere passive fillers, these residues function as natural surfactants taking advantage of the amphiphilic nature of the lipids and phenolic compounds present in coffee to overcome a major limitation in nanotechnology: particle agglomeration. This bio-based intervention not only improved layer-to-layer adhesion in full-flow 3D printing (FGF), but was also crucial for structuring the conductive network, increasing the electrical conductivity from 0.2 × 10^−1^ to 2.6 × 10^−1^ S/cm and enabling the fabrication of functional strain gauges.

Bio-based additives have evolved into strategic tools for programming polymer lifecycles, creating a functional dichotomy between extending durability and accelerating disintegration. Regarding preservation, Shnawa [76] utilized calcium humates to thermally stabilize PVC, while Moreira et al. [77] employed *Acacia mearnsii* tannins to retard biodegradation in PLA/starch composites via antimicrobial action. Conversely, Wolf et al. [78] and Soydal et al. [79] focused on disintegration catalysts. Wolf et al. introduced an alginate-based ionic “switch” that triggers fracture upon environmental cation exposure. This mechanism is likely driven by an ion-exchange process that promotes localized swelling and internal mechanical stress, facilitating polymer chain scission. This approach contrasts with Soydal’s use of agro-residues to passively enhance hydrophilicity. Additionally, Vogelgesang et al. [80] demonstrated that depolymerized hemicellulose acts as a spectroscopic tracer, establishing a “digital identity” for automated sorting.

Preservation strategies demonstrate high efficiency at low dosages without compromising mechanics. Shnawa [76] reported that only 3.8 wt% (4 phr) of calcium humate increased the PVC initial decomposition temperature (Ti5%) from 270 °C to 290 °C, outperforming zinc variants that induced “zinc burning.” Similarly, Moreira et al. [77] found that 1 wt% tannin not only delayed biodegradation (reducing 60-day mass loss from 40% to 30%) but also reinforced the matrix, increasing tensile strength by 40.4% (from 2.08 to 2.92 MPa).

In contrast, degradation accelerators often present a trade-off between loading and performance. Soydal et al. [79] required a 50 wt% loading of sour cherry waste to achieve 40% mass loss over 12 months, which reduced mechanical strength from 4.60 to 2 MPa. Wolf et al. [78] mitigated this issue via a chemical-trigger mechanism; while soil biodegradation increased modestly (to 5%), molecular analysis revealed extensive chain scission (Mw drop from 2.26 × 10^5^ to 8.50 × 10^4^ g/mol), proving efficient breakdown without volumetric overloading. Finally, regarding identification, Vogelgesang et al. [80] confirmed the viability of micro-dosages, achieving 95–99.8% sorting accuracy with 0.5–1% hemicellulose markers, noting that concentrations below 0.1% are susceptible to signal noise.

Synthesizing these diverse applications reveals a critical shift: bio-based additives now act as active, programmable agents rather than passive fillers. Across mass transport, crystallization, and lifecycle management, the unifying principle is exploiting the inherent chemical complexity of biomass, such as specific polar domains and amphiphilicity. The primary takeaway for future material design is maximizing functional efficiency at minimal loadings. While bulk physical modifications often require high volumetric fractions that compromise structural integrity, targeted molecular interventions achieve profound microstructural control at concentrations typically below 5 wt%. Consequently, the next frontier in biopolymer engineering relies on molecular programming to create smart, self-regulating materials fully integrated into circular economy frameworks.

Despite the robust findings, some limitations must be acknowledged. Regarding the evidence, most studies are conducted at a laboratory scale, which may not fully capture the complexities of industrial-scale processing or long-term migration in diverse environments. Regarding the review process, the search was limited to the Scopus database, which, although comprehensive, might have excluded specific regional technical reports or studies published in non-indexed journals. However, the systematic nature of this review ensures a high level of transparency and reproducibility, providing a strategic roadmap for future research focusing on the scalability of green synthesis and standardized life-cycle assessments.

## 4. Conclusions and Future Perspectives

The transition from fossil-based to bio-based polymer additives has evolved from direct substitution strategies to the engineering of multifunctional, high-performance molecules. This review highlights that the most significant advances in the 2023 to 2026 period lie in the development of “all-in-one” additives, such as phosphorylated lignin and chemically modified phytates, which simultaneously provide plasticity, flame retardancy, and antioxidant protection without compromising the material’s biodegradability.

Despite the exponential growth in publications, critical challenges remain. Bibliometric and experimental analyses indicate that while low-dosage additives (typically <5 wt%) have achieved competitive performance in stabilization and nucleation, high-loading applications (fillers and flame retardants) still face limitations regarding interfacial compatibility and processing viscosity. The emerging concept of “reactive windows” for compatibilizers and the use of biomimetic structures (e.g., terpenes and tannins) point toward a new generation of additives that are chemically integrated into the polymer network rather than physically blended.

Future research must prioritize three key areas: (1) the scalability of solvent-free enzymatic synthesis to reduce cost; (2) the rigorous assessment of migration and toxicity of bio-based derivatives in food-contact scenarios, ensuring they meet safety standards equivalent to or better than their fossil counterparts; and (3) the design of additives that not only improve performance during use but actively trigger or facilitate recycling and biodegradation at the end of life. Beyond technical goals, the socio-economic pillar of sustainability must be strengthened; utilizing localized biomass residues can mitigate the geopolitical dependencies and supply chain vulnerabilities inherent to fossil-based resources, especially in contexts of global instability and resource scarcity. Bridging the gap between laboratory synthesis and industrial extrusion requirements will be the definitive step toward a truly holistic and sustainable plastics economy.

## Figures and Tables

**Table 1:** Representative chemical nomenclature, classification, and structural representation of selected bio-based functional additives.

Additive (Abbrev.)	Name	Class	Structural Representation
GT	Glycerol Trilevulinate	Plasticizer	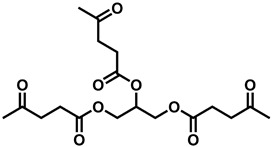
TPAG	Triphenyl Acetic Glyceroate	Multifunctional plasticizer	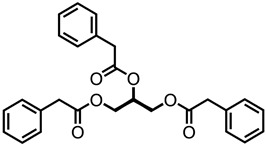
PMBL	Phosphaphenanthrene groups-containing maleic acid 2-(2-n-butoxyethoxy) ethanol lactate ester.	Plasticizer + Flame Retardant	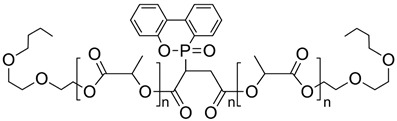
VA8-P	Vanillic acid-based phosphate ester	Aromatic hybrid: Plasticizer + Flame Retardant	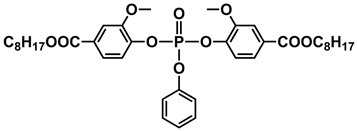
U-DC	Urushiol-derived phosphonate	Flame Retardant	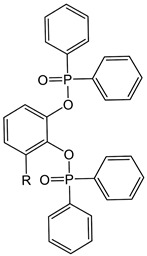
PLO	Poly(limonene oxide)	Plasticizer (oligomer behavior)	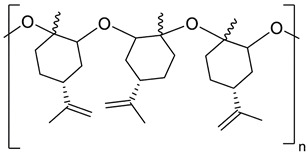
PA1PEA12	Phytic acid phenethylamine	Flame Retardant (FR)	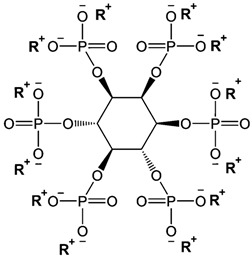
DBI	Dibutyl itaconate	Plasticizer/Compatibilizer	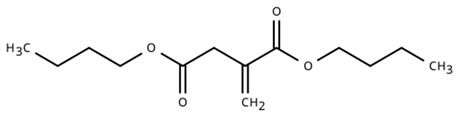
DFFD	2,5-diformylfuran dioxime	Surface Agent/Antimicrobial	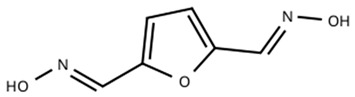

**Table 2:** Comparative overview of recently reported bio-based plasticizers: synthesis routes, biomass origin, and experimental dosages.

Plasticizer	Raw Materials	Synthesis Route	Dosage (wt%)	Ref.
Glycerol Trilevulinate (GT)	Levulinic acid (LA) and Glycerol (GLY)	Single-step: Solvent-free esterification (110 °C, 16 h)	2.5, 5, and 10	[28]
Coffee Oil Epoxide (COE)	Spent coffee grounds	Extraction of lipid fractions from spent coffee grounds followed by epoxidation	0.3	[29]
Cardanol-based Polyesters	Cardanol-derived diol, dimethyl succinate (DMS), and diethyl adipate (DMA)	Two-step enzymatic polycondensation: (1) Solvent-free reaction (85 °C, 6 h); (2) Vacuum step (18 h) to increase molecular weight	2, 5, and 10	[30]
Poly[di(ethylene glycol) adipate]	Linear aliphatic polyester	Melt-blending (Haake mixer, Thermo Fisher Scientific, Waltham, MA, USA, 185 °C, 50 rpm for 3 min) followed by compression molding.	10 and 15	[32]
Esters of PTA and MSA (TEPTC, TBPTC, TOPTC, DBMS)	Citric acid derivatives (PTA/MSA) and alcohols (Ethanol, Butanol, Octanol)	Reductive removal of CA hydroxyl group followed by Fischer esterification (16–20 h, H_2_SO_4_)	1, 2.5, and 5	[31]
Polylimonene oxide (PLO)	(+)-Limonene oxide (LO)	Ring-opening polymerization (ROP): Bulk polymerization at room temperature (25 °C) using aluminium-based catalysts.	5, 10, 15, and 20	[33]
Glycerol-oligo(lactic acid) bioresins (S3 and S7)	Lactic acid (LA) and Glycerol (GLY)	Single-step polycondensation: Solvent-mediated (toluene) reaction at 110 °C (24 h) using PTSA catalyst and Dean-Stark trap	10 and 20	[34]
TPAG (Triphenyl Acetic Glyceroate)	Solvent-free esterification of Glycerol and Phenylacetic acid	Single step: Direct solvent-free esterification	5, 9, and 17	[35]
PBCL (Phosphorylated Crotonic acid Lactate ester)	L-lactic acid, crotonic acid, and DOPO (phosphaphenanthrene group)	Sequential esterification of L-lactic acid and crotonic acid with alcohol, followed by DOPO addition	17	[36]
VA8-P	Vanillic acid (lignin-derived), ethylhexanol and phenyl dichlorophosphate	Esterification of vanillic acid with ethylhexanol followed by phosphorylation with PDCP	17, 29, and 38	[37]
PMBL	Synthesis from L-lactic acid, 2-(2-n-butoxyethoxy) ethanol, maleic acid, and DOPO	Sequential esterification of L-lactic acid and alcohol with maleic acid, followed by DOPO addition	17, 23, and 29	[38]

**Table 3:** Comparative overview of natural antioxidants in diverse polymer matrices.

Natural Antioxidant	Polymer Matrix	Concentration (wt%)	Main Differentiator	Reference
Organosolv lignin from beech wood	Epoxi (DGEBA)	3–16.6	Acts as a reactive curing agent (solvent-free).	[45]
Eucalyptus Lignin	PP (Recycled)	0.05–0.5	Superiority over synthetics at low doses.	[46]
Lignin from pine wood	PCL	10–100	Use of green solvent for lignin extraction.	[47]
Grape Pomace	PBS	1–20	Stabilization influenced by climatic factors.	[48]
Aronia and Lima Beans	PHBV	2.5–7	Thermal stabilization in continuous processing.	[49]
Tannins	PHBV	0.99–9.09	Smart packaging with ammonia sensor.	[50]

## Data Availability

No new data were created or analyzed in this study.

## References

[1] Kowalczuk M. (2023). Polymer materials—Challenges and hope. Front. Soft Matter.

[2] Harun-Ur-Rashid M., Imran A.B. (2024). Emerging Trends in Engineering Polymers: A Paradigm Shift in Material Engineering. Recent Prog. Mater..

[3] Dokl M., Copot A., Krajnc D., Van Fan Y., Vujanović A., Aviso K.B., Tan R.R., Kravanja Z., Čuček L. (2024). Global projections of plastic use, end-of-life fate and potential changes in consumption, reduction, recycling and replacement with bioplastics to 2050. Sustain. Prod. Consum..

[4] Morris B.A. (2024). A perspective on additives for flexible packaging. Vinyl Addit. Technol..

[5] Malík J., Kröhnke C. (2006). Polymer stabilization: Present status and possible future trends. C. R. Chim..

[6] Culleré L., Sangüesa E., Lomba L., Ribate M.P., Zuriaga E., García C.B. (2025). A Systematic Review: Migration of Chemical Compounds from Plastic Material Containers in Food and Pharmaceutical Fields. J. Xenobiotics.

[7] Qadeer A., Anis M., Warner G.R., Potts C., Giovanoulis G., Nasr S., Archundia D., Zhang Q., Ajmal Z., Tweedale A.C. (2024). Global environmental and toxicological data of emerging plasticizers: Current knowledge, regrettable substitution dilemma, green solution and future perspectives. Green Chem..

[8] Lee Y.-M., Lim Y., Kang S., Kim S. (2025). Migration of phthalate and non-phthalate plasticizers from polyvinyl chloride (PVC) materials. Sci. Total Environ..

[9] Korkmaz S.D., Küplülü Ö., Aral G.I., Şeker M.E. (2023). Migration of phthalates from plastic packages into dairy products. Kafkas Univ. Vet. Fak. Derg..

[10] Seref N., Cufaoglu G. (2025). Food Packaging and Chemical Migration: A Food Safety Perspective. J. Food Sci..

[11] Palandri L., Monti M., Scasserra M.R., Lugli C., Fasano M., Lucaccioni L., Righi E. (2026). Regulatory framework of phthalates and two common alternatives: A review of the European Union legislation. Int. J. Hyg. Environ. Health.

[12] European Chemicals Agency (2023). Regulatory Strategy for Flame Retardants.

[13] Velasquez S.T.R., Hu Q., Kramm J., Santin V.C., Völker C., Wurm F.R. (2025). Plastics of the Future? An Interdisciplinary Review on Biobased and Biodegradable Polymers: Progress in Chemistry, Societal Views, and Environmental Implications. Angew. Chem. Int. Ed..

[14] De Boer J., Harrad S., Sharkey M. (2024). The European Regulatory Strategy for flame retardants—The right direction but still a risk of getting lost. Chemosphere.

[15] Marturano V., Marotta A., Salazar S.A., Ambrogi V., Cerruti P. (2023). Recent advances in bio-based functional additives for polymers. Prog. Mater. Sci..

[16] Sampaio M.S., Lima M.T., Wojcieszak R., Itabaiana I. (2025). Recent Advances in the Synthesis of Bio-Based Polymers: Toward a More Sustainable Future. ChemistrySelect.

[17] Rajendran D.S., Venkataraman S., Jha S.K., Chakrabarty D., Kumar V.V. (2024). A review on bio-based polymer polylactic acid potential on sustainable food packaging. Food Sci. Biotechnol..

[18] Chen M., Guo Q., Yuan Y., Li A., Lin B., Xiao Y., Xu L., Wang W. (2025). Recent Advancements of Bio-Derived Flame Retardants for Polymeric Materials. Polymers.

[19] Fan T., Yan Z., Huang W., Feng W., Bai Y., Feng C., Wu F. (2025). A comprehensive review of contents, toxic effects, metabolisms, and environmental behaviors of brominated and organophosphorus flame retardants. J. Hazard. Mater..

[20] Leite-Barbosa O., Pinto C.C.D.O., Leite-da-Silva J.M., De Aguiar E.M.M.M., Veiga-Junior V.F. (2024). Polymer Composites Reinforced with Residues from Amazonian Agro-Extractivism and Timber Industries: A Sustainable Approach to Enhancing Material Properties and Promoting Bioeconomy. Polymers.

[21] Cakir Yigit N., Karagoz I. (2023). A review of recent advances in bio-based polymer composite filaments for 3D printing. Polym.-Plast. Technol. Mater..

[22] Olonisakin K., Mohanty A.K., Thimmanagari M., Misra M. (2025). Recent advances in biodegradable polymer blends and their biocomposites: A comprehensive review. Green Chem..

[23] Vasile C., Tantaru G., Creteanu A. (2025). Recent Insights into the Research of (Bio)Active Additives for Advanced Polymer Materials. Polymers.

[24] Schick S., Heindel J., Groten R., Seide G.H. (2024). Overcoming Challenges in the Commercialization of Biopolymers: From Research to Applications—A Review. Polymers.

[25] Wang M., Yin G.-Z., Yang Y., Fu W., Palencia J.L.D., Zhao J., Wang N., Jiang Y., Wang D.-Y. (2023). Bio-based flame retardants to polymers: A review. Adv. Ind. Eng. Polym. Res..

[26] Ridard H., Duvigneau J., Mayer-Gall T., Ali W., Wurm F.R. (2024). Biobased Flame-Retardant Polylactic Acid Foams through Lignin-Based Nanocarriers Encapsulating Deoxyribonucleic Acid. ACS Sustain. Chem. Eng..

[27] Leite-Barbosa O., Oliveira M.F.L.D., Braga F.C.F., Monteiro S.N., Oliveira M.G.D., Veiga-Junior V.F. (2024). Impact of Buriti Oil from Mauritia flexuosa Palm Tree on the Rheological, Thermal, and Mechanical Properties of Linear Low-Density Polyethylene for Improved Sustainability. Polymers.

[28] Togliatti E., Lenzi L., Degli Esposti M., Castellano M., Milanese D., Sciancalepore C., Morselli D., Fabbri P. (2024). Enhancing melt-processing and 3D printing suitability of polyhydroxybutyrate through compounding with a bioplasticizer derived from the valorization of levulinic acid and glycerol. Addit. Manuf..

[29] Ghosh R., Zhao X., Vodovotz Y. (2025). Rheological Behavior and Mechanical Performance of Poly(3-hydroxybutyrate-co-3-hydroxyvalerate)/Natural Rubber Blends Modified with Coffee Oil Epoxide for Sustainable Packaging Applications. Polymers.

[30] Vallin A., Ferretti F., Campaner P., Monticelli O., Pellis A. (2023). Environmentally Friendly Synthesis of Cardanol-Based Polyesters and Their Application as Poly(lactic acid) Additives. ACS Sustain. Chem. Eng..

[31] De Bruyne A., Gómez K.C., O’rOurke G., Denayer M., Vekeman J., De Proft F., Stuyck W., Leinders J., Van Puyvelde P., De Vos D. (2024). New Tricarboxylate Plasticizers for Use in Polylactic Acid: Synthesis, Thermal Behavior, Mechanical Properties and Durability. J. Polym. Environ..

[32] Montes M.L.I., Malbos L.B., Hankovits M.I., Giacomini A., D’aMico D.A., Seoane I.T., Manfredi L.B., Cyras V.P. (2025). Effect of biobased polymeric plasticizer on the properties of polylactic acid/polyhydroxybutyrate blends for biodegradable packaging. Polym. Bull..

[33] Palenzuela M., Vega J.F., Souza-Egipsy V., Ramos J., Rentero C., Sessini V., Mosquera M.E.G. (2023). Insight into the melt-processed polylimonene oxide/polylactic acid blends. Polym. Chem..

[34] Alijanian S., Zohuriaan-Mehr M.J., Esmaeilzadeh M., Salimi A., Razavi-Nouri M. (2024). Glycerol-oligo(lactic acid) bioresins as fully biobased modifiers for poly(Lactic acid): Synthesis, green chemistry metrics, and the modified PLA film Properties. J. Polym. Environ..

[35] Ferri M., Lenzi L., Degli Esposti M., Martellosio L., Benítez J.J., Hierrezuelo J., Grifé-Ruiz M., Romero D., Guzmán-Puyol S., Heredia-Guerrero J.A. (2025). Triphenyl Acetic Glyceroate as a sustainable multifunctional additive for developing transparent, biodegradable, and flexible polylactide green alternative to polyethylene-based films for food packaging. Chem. Eng. J..

[36] Hou B., Wang Y., Gong T., Wang R., Huang L., Li B., Li J. (2024). Fabrication of highly efficient biodegradable oligomeric lactate flame-retardant plasticizers for ultra-flexible flame-retardant poly (lactic acid) composites. Chem. Eng. J..

[37] Qian B., Wang W., Zhu H., Zhang J., Wu M., Liu J., Wu Q., Yang J. (2023). Synthesis, characterization and performance evaluation of a flame retardant plasticizer for poly(vinyl chloride) derived from biobased vanillic acid. Chem. Eng. J..

[38] Sun Y., Hou B., Liu B., Shen H., Zhang M. (2025). Fabrication of biodegradable phosphorus-containing flame-retardant plasticizer derived from L-lactic acid toward simultaneously enhancing the toughness, flame retardancy and transparency of flexible polyvinyl chloride. Polym. Degrad. Stab..

[39] Li P.-K., Liu I.-T., You J.-L., Liao Y.-C. (2026). A sustainable vitrimer from depolymerized lignin: Synthesis and reworkable application. Int. J. Biol. Macromol..

[40] Majka T.M., Al Nakib R., Menceloglu Y.Z., Pielichowski K. (2025). The Influence of Lignin Derivatives on the Thermal Properties and Flammability of PLA+PET Blends. Materials.

[41] Zhang Y., Qi G., Xu Y., Zhang X., Yang W., Xu P. (2025). Fully bio-based flame retardancy in polyhydroxyalkanoates: Sustainable engineering through phytic acid-derived additives. Int. J. Biol. Macromol..

[42] Liang K.-X., Chen J.-L., Lin Y.-C., Bai W.-B., Jian R.-K. (2024). Natural resin-derived urushiol-based phosphorylation derivative towards flame-retardant and mechanically strong epoxy resin. Polym. Degrad. Stab..

[43] Cao X., Tian X., Huang Y., Zhang L., Ni Y., Wang Y.-Z. (2025). H3PO3-protonated chitosan enabling flame-retardant and antibacterial PVA composite films with high strength and toughness through multiple H-bonds and interlocking interfaces. Chin. Chem. Lett..

[44] Aaddouz M., Laoutid F., Mariage J., Yada B., Toncheva A., Lazko J., Azzaoui K., Sabbahi R., Mejdoubi E., Saeb M.R. (2025). Mechanochemistry for the Synthesis of a Sustainable Phosphorus/Potassium Tannic Acid Flame-Retardant Additive and Its Application in Polypropylene. ACS Sustain. Chem. Eng..

[45] Pappa C.P., Torofias S., Triantafyllidis K.S. (2023). Sub-Micro Organosolv Lignin as Bio-Based Epoxy Polymer Component: A Sustainable Curing Agent and Additive. ChemSusChem.

[46] Babetto A.C., Garcia Gonçalves L.M., Silva E.A., Lima Neto B.S., Morandim-Giannetti A.D.A., Prado Bettini S.H. (2025). Can all lignins act as an antioxidant for polypropylene?. Polym. Test..

[47] Melro E., Duarte H., Antunes F.E., Valente A.J.M., Romano A., Medronho B. (2025). Enhancing Polycaprolactone with Levulinic Acid-Extracted Lignin: Toward Sustainable Bio-Based Polymer Blends. J. Compos. Sci..

[48] Hiller B.T., Schübel L., Rennert M., Krieg D., Nase M., Puch F. (2025). Study of Wine Grape Pomaces from Different Vintages Regarding Their Use as Reliable Sustainable Antioxidants in Biobased Poly(Butylene Succinate). J. Polym. Environ..

[49] Rusko A., Boutigny M., Ruckdäschel H., Bonten C. (2026). Natural Antioxidants as Thermal Stabilizers in Fully Biobased PHBV Compounds. J. Appl. Polym. Sci..

[50] Ferri M., Papchenko K., Degli Esposti M., Tondi G., De Angelis M.G., Morselli D., Fabbri P. (2023). Fully Biobased Polyhydroxyalkanoate/Tannin Films as Multifunctional Materials for Smart Food Packaging Applications. ACS Appl. Mater. Interfaces.

[51] Srisuwanno T., Johns J., Bascucci C., Clemens F., Nakaramontri Y. (2025). Effect of bio-based epoxidized natural rubber as a compatibilizer in thermoplastic polyurethane/natural rubber blends: Physical characterization and 3D printing behavior. Express Polym. Lett..

[52] Morinval A., Avérous L. (2024). Synthesis by Fast Thiol-Michael Click Addition of Biodegradable and Potentially Fully Biobased Architectures Based on Starch and Polyester, Toward Sustainable and Performing Multiphase Systems. ACS Sustain. Chem. Eng..

[53] Arslan Ç., Tayfun Ü., Yılmaz V.M., Delikanlı N.E. (2026). Comprehensive Investigations of Injection Molding and Additive Manufacturing on Mechanical and Structural Characteristics of Polylactide Composites Loaded with Basalt Fiber Involving Bio-Based Compatibilizers. Vinyl Addit. Technol..

[54] Jing Y., Zhang Y., Bai X., Qiu Y., Cong F., Yu L., Gao G., Huang J., Leng S., Wang N. (2025). Optimizing the performance of poly(lactic acid)/poly (butylene adipate-co-terephthalate) blends: Improved toughness, thermal resistance, and degradation property. Int. J. Biol. Macromol..

[55] Wu X., Ginoux G., Paux J., Allaoui S. (2025). Damage and fracture studies of continuous flax fiber-reinforced composites 3D printed by in-nozzle impregnation additive manufacturing. Int. J. Damage Mech..

[56] Frasca S., Katsiotis C.S., Henrik-Klemens Å., Larsson A., Strømme M., Lindh J., Galkin M.V., Gising J. (2024). Compatibility of Kraft Lignin and Phenol-Organosolv Lignin with PLA in 3D Printing and Assessment of Mechanical Recycling. ACS Appl. Polym. Mater..

[57] Ilhan R., Gumus O.Y., Lekesiz H. (2025). Biodegradable Nanocomposite Filament Based on PLA/PCL/CNCS for FDM 3D Printing: Production, Characterization and Printability. Polym. Compos..

[58] Mandala R., Anjaneya Prasad B., Akella S., Mallaiah M., Thapliyal S., Chandra Bose S. (2025). 3D Printing of PLA/Groundnut Shell-Derived Biocarbon Composite Using a Pellet Extruder. Recent Advances in Additive Manufacturing.

[59] Sanchez-Sobrado O., Tiniakos A.F., Abalde R., Rivas M., Grigoropoulos A., Nikolaou A., Zoikis-Karathanasis A., Deligkiozi I., Losada R. (2026). Improved properties of high-density polyethylene by integrating high content of bio-fillers based on green nanolignin for applications in plastic industry. Compos. Part C Open Access.

[60] Banpean A., Hararak B., Winotapun C., Wijaranakul P., Kitchaicharoenporn S., Naimlang S. (2023). Lignin nanoparticles as sustainable biobased nucleating agents of poly(L-lactic acid): Crystallization behavior and effect of particle sizes. J. Mater. Sci..

[61] Schulz N.M., Papadopoulos L., Hagenlocher L., Gohla A., Bikiaris D.N., Robert T. (2025). Wood and olive kernel flour as reinforcement for itaconic acid-based UV-curing additive manufacturing material. React. Funct. Polym..

[62] Romero-Ocaña I., Herrera M., Fernández-Delgado N., Molina S.I. (2025). Potential Use of Residual Powder Generated in Cork Stopper Industry as Valuable Additive to Develop Biomass-Based Composites for Injection Molding. J. Compos. Sci..

[63] Rus A.Z.M., Wahab H.A., Saif Y., Marsi N., Zaliran M.T., Alamshah M.H., Mariza I., Rus S.M., Al-Alimi S., Zhou W. (2025). Morphological and acoustical characterization of UV-irradiated foam composites from cooking oil and wood flake. J. Polym. Res..

[64] Matsumoto Y., Abdellatif M.M., Nomura K. (2024). Polymer composites of biobased aliphatic polyesters with natural abundant fibers that improve the mechanical properties. J. Mater. Cycles Waste Manag..

[65] Yeh Y.-S., Wei Y.-T., Chang J.-S., Ng I.-S. (2025). Fabrication and characterization of microalgae-based additive ethylene vinyl acetate composites as sustainable bioplastics. Int. J. Biol. Macromol..

[66] Khenari A.R., Abedini R., Hassanzadeh H. (2026). From pepper to polymer: Green modification of PMP membranes for efficient CO_2_ separation. Polym. Bull..

[67] Al Marri S.H., Manawi Y., Simson S., Lawler J., Kochkodan V. (2025). Novel Ultrafiltration Polyethersulfone Membranes Blended with Carrageenan. Polymers.

[68] Li S., Svedström K., Lepo A. (2024). Biowaxes Used as a Barrier Formulation Material in Coating Fibre-Based Substrates. Packag. Technol. Sci..

[69] Yolacan O., Deniz S. (2025). Effects of different biopolymers and additives on mechanical and barrier properties of Poly(3-hydroxybutyrate-co-3-hydroxyvalerate) blend films. J. Dispers. Sci. Technol..

[70] Marchi P., Wang W., Puig C., Martin A., Crovetto T., Labidi J., Riva R., Cavallo D., Moni L. (2023). Synthesis of symmetric bis-α-ketoamides from renewable starting materials and comparative study of their nucleating efficiency in PLLA. RSC Adv..

[71] Carrero K.C.N., Herrero M., Alonso L.E., Lizalde-Arroyo F., Salmazo L.O., Merino J.C., Rodríguez-Pérez M.Á., Barajas J.M.P. (2024). Biopolyamide composites for fused filament manufacturing: Impact of fibre type on the microstructure and mechanical performance of printed parts. Prog. Addit. Manuf..

[72] Peng S., Luo L., Wu R., Yi Z., Wuliu Y., Shi P., Zhu J., Feng J., Liu Y. (2023). Study on scratch behavior and application of a novel cardanol-based additive in polypropylene. Tribol. Int..

[73] Yang G., Lu G., Liu Z., Tian S., Ma Z., Zhang Y. (2025). Enhancement of the Excellent Properties of an Intrinsically Antimicrobial Poly(Oxime-Urethane) Coating with a Bio-Based Furan Chain Extender. Macromol. Rapid Commun..

[74] Gaddam S.K., Boddu V.R., Boga K., Bhatt H.K., Das P., Pinjari S.D., Qi X., Gaddam R.R., Arukula R. (2025). Maleinized Vegetable Oil: A Green Approach for Sustainable Polyamide Composites. Polym. Adv. Technol..

[75] Lage-Rivera S., Ares-Pernas A., Dopico-García M.S., Costa C.M., Pereira N., Lanceros-Mendez S., Abad M. (2025). Using Lignin and Spent Coffee Grounds as Bio-Additives in 3D-Printable Poly Lactic Acid/Multi-Walled Carbon Nanotube Composites for Advanced Electronic Applications. Polym. Compos..

[76] Shnawa H.A. (2023). Synthesis and evaluation of calcium and zinc humates for stabilization of poly(Vinyl Chloride) and study their self-synergistic effect. J. Polym. Res..

[77] Moreira A.A., de Carvalho F.A., Bilck A.P., de Paula M.T., Mali S., Yamashita F., de Oliveira A.L.M. (2023). Tannin improves the processability and delays the biodegradability of poly (lactic acid)-starch-based thermoset materials produced by injection molding made with renewable compounds. J. Appl. Polym. Sci..

[78] Wolf P., Helberg J., Zollfrank C. (2025). Switchable ion-induced (bio)degradation of a novel polylactic acid composite including microfibrillated cellulose and calcium alginate. Polym. Degrad. Stab..

[79] Soydal U., Ahmetli G., Yıldırım M., Işık M., Okcuoglu M.C., Bul M.M. (2024). Production and characterization of novel biodegradable films using fruit industrial waste and aloe vera gel. Polym. Bull..

[80] Vogelgesang M., Trojanowski B., Hanstein S., Zuge A., Benner W., Ionescu E. (2025). Detection of bio-based additives in plastics using NIR data: Opportunity for bio-based markers. Proceedings of the OCM 2025, 7th International Conference on Optical Characterization of Materials, Karlsruhe, Germany, 26–27 March 2025.

